# Ctp1 and Yhm2: Two Mitochondrial Citrate Transporters to Support Metabolic Flexibility of *Saccharomyces cerevisiae*

**DOI:** 10.3390/ijms25031870

**Published:** 2024-02-03

**Authors:** Graziana Assalve, Paola Lunetti, Vincenzo Zara, Alessandra Ferramosca

**Affiliations:** 1 Department of Biological and Environmental Sciences and Technologies, University of Salento, 73100 Lecce, Italy; graziana.assalve@unisalento.it (G.A.); paola.lunetti@unisalento.it (P.L.); vincenzo.zara@unisalento.it (V.Z.); 2 Department of Experimental Medicine, University of Salento, 73100 Lecce, Italy

**Keywords:** citrate, intermediary metabolism, metabolite carrier, mitochondria, metabolism, subcellular compartments

## Abstract

Differently from higher eukaryotic cells, in the yeast *Saccharomyces cerevisiae* there are two mitochondrial carrier proteins involved in the transport of citrate: Ctp1 and Yhm2. Very little is known about the physiological role of these proteins. Wild-type and mutant yeast strains deleted in *CTP1* and *YHM2* were grown in media supplemented with a fermentable (glucose) or a nonfermentable (ethanol) carbon source. To assess changes in Ctp1 and Yhm2 mRNA expression levels, real-time PCR was performed after total RNA extraction. In the wild-type strain, the metabolic switch from the exponential to the stationary phase is associated with an increase in the expression level of the two citrate transporters. In addition, the results obtained in the mutant strains suggest that the presence of a single citrate transporter can partially compensate for the absence of the other. Ctp1 and Yhm2 differently contribute to fermentative and respiratory metabolism. Moreover, the two mitochondrial carriers represent a link between the Krebs cycle and the glyoxylate cycle, which play a key role in the metabolic adaptation strategies of *S. cerevisiae*.

## 1. Introduction

The yeast *Saccharomyces cerevisiae* is one of the most widely used model organisms for investigating the main cellular functions that are conserved in more complex organisms, including humans. This unicellular organism can adapt its metabolism in response to fluctuations in nutrient availability to meet the energy demand and survive [1,2,3].

For instance, in the presence of high quantities of glucose, the yeast prefers fermentation as the central route to generate ATP and this effect is known as the Crabtree effect [4]. These growth conditions are characterized by an increase in glycolytic flux and repression of the transcription of genes involved in mitochondrial respiration, in the catabolism of alternative carbon sources such as fatty acids, as well as in anabolic pathways such as gluconeogenesis, glycogen synthesis, and glyoxylate cycle [5,6].

On the other hand, when glucose is unavailable, *S. cerevisiae* can produce metabolic energy utilizing a wide variety of alternative carbon sources, including nonfermentable compounds such as ethanol [7,8]. Growth of *S. cerevisiae* with ethanol requires its oxidative breakdown to generate not only ATP, but also cellular biomass through the formation of sugar phosphates and other carbon metabolites, such as storage carbohydrates, lipids, and amino acids [9].

Therefore, this metabolic flexibility found in yeast depends on the specific substrates available during growth conditions. In this respect, mitochondrial carrier proteins play a key role because they transport a variety of substrates across the inner membrane of the organelles, thus connecting mitochondria with cytosolic reactions [10,11,12]. Among other substrates, citrate is a key metabolic intermediate involved in several pathways and, therefore, the flux of this molecule between different cellular compartments is strictly regulated.

In *S. cerevisiae* cells, the synthesis of citrate depends on the activity of two citrate synthase isoforms located into mitochondria (CIT1) and peroxisomes (CIT2), which catalyze the condensation of oxaloacetate with acetyl-CoA [13,14]. Mitochondrial citrate generated by CIT1 is an intermediate of the Krebs cycle, where it is further catabolized to generate NADH and FADH_2_. These reduced coenzymes deliver electrons to the respiratory complexes of the mitochondrial transport chain to produce ATP. Extramitochondrial citrate generated by CIT2 is an intermediate of the glyoxylate pathway, which occurs in cytosol and peroxisomes, thus allowing yeast cells to make a net conversion of fatty acids to carbohydrates. Citrate can be therefore transported out and into mitochondria by using specific carrier proteins located in the mitochondrial inner membrane [11,15]. Therefore, an additional source of cytosolic citrate might be the export of mitochondrially produced citrate [15,16,17]. In this context, the role of citrate transporters in metabolic adaptation upon stress conditions has been previously reported in yeast. In particular, an increase in citrate export from both peroxisomes and mitochondria has been hypothesized in stress conditions, with subsequently increased production of the cytosolic NADPH [18].

Differently from higher eukaryotic cells, where there is only one mitochondrial citrate transporter [19], in *S. cerevisiae* there are two carrier proteins involved in the transport of citrate across the inner mitochondrial membrane: Ctp1 and Yhm2. It has been proposed that the first one (Ctp1) transports citrate from mitochondria to cytosol, or vice versa, in exchange for another tricarboxylate. Alternatively, malate can be exchanged with citrate, although it can be transported to a considerably lesser extent [16,20]. Castegna et al. proposed that the second one (Yhm2) catalyzes the citrate/α-ketoglutarate shuttle between mitochondria and cytosol, giving an important contribution to the regeneration of cytosolic NADPH, which is required for biosynthetic and antioxidant reactions [17].

Very little is known about the yeast mitochondrial citrate transporter Ctp1, which can be considered the corresponding isoform of citrate transport protein characterized in higher eukaryotic cells [16]. Even less information is available on Yhm2, which exports citrate from and imports α-ketoglutarate into the mitochondria, causing net export of reducing equivalents in the form of NADPH. Interestingly, this protein also associates with mitochondrial nucleoids and has a role in the replication and segregation of the mitochondrial genome [17,21].

Studies on the structural and functional characterization of these two carrier proteins have been carried out by expressing the corresponding genes in *E. coli,* reconstituting the gene products into liposomes, and testing the transport activity in the presence of selected substrates [10]. However, the correlation between the in vitro activities of the citrate carriers of *S. cerevisiae* and their specific physiological roles in in vivo reactions remains to be ascertained. Thus, there is no direct evidence to determine whether and when the citrate transport occurs as catalyzed by one or two carriers, although it appears that Ctp1 cannot substitute the antioxidant function of Yhm2 [17].

In this study, we tried to understand the role of Ctp1 and Yhm2 in *S. cerevisiae* physiology by investigating a possible function of these proteins in the adaptation of yeast metabolism in response to specific growth conditions. To this end, growth media containing either fermentable (glucose) and nonfermentable (ethanol) carbon sources has been used to culture wild-type and mutant yeast strains lacking Ctp1 (∆*ctp1*) or Yhm2 (∆*yhm2*).

Analysis of the expression of the two citrate transporters during yeast growth under different experimental conditions led us to attribute a key role to Ctp1 and Yhm2 in the metabolic adaptation strategies of *S. cerevisiae* in response to nutritional changes.

## 2. Results

### 2.1. Glucose and Ethanol Metabolism during S. cerevisiae Growth

The growth of *S. cerevisiae*, which can be measured by growth curves and serial dilutions for spot testing, is a valuable tool for investigating yeast physiological mechanisms.

Yeast growth is usually divided into well-defined phases: the lag phase, the exponential phase, and the stationary phase. During the lag period, the cell number does not change, whereas in the exponential phase, cell division occurs, leading to a rapid increase in cell numbers. Finally, the stationary phase is characterized by cell cycle arrest.

These growth phases depend on the nutrient availability of the surrounding medium at different stages. In fact, the stationary phase is characterized by a general depletion of nutrients and an excess of potentially harmful molecules accumulated during the exponential phase.

**Figure 1:**
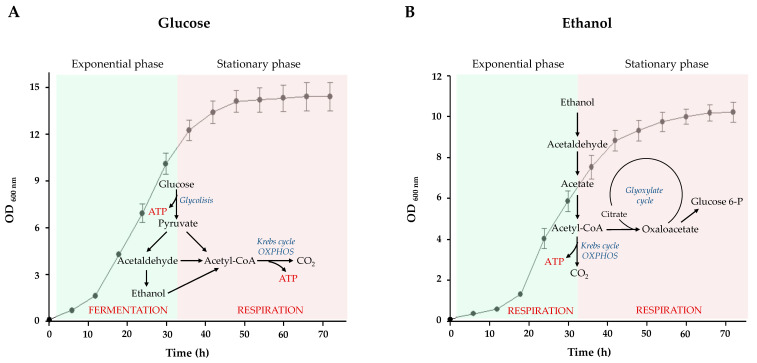
Growth curves of the wild-type yeast cells and pathways for ATP synthesis in the presence of fermentable (glucose) and nonfermentable (ethanol) carbon sources. Wild-type cells were inoculated in liquid glucose- or ethanol-supplemented medium and the OD values at 600 nm after the indicated growth times are reported in the figure as mean ± standard deviation (SD). (**A**) In the presence of glucose, *S. cerevisiae* cells in the exponential phase show a fermentative metabolic phenotype, characterized by a high glycolytic flux, ethanol production, and a low oxygen uptake rate. In the stationary phase, yeast cells decreased glycolytic flux, switching their metabolism towards a respiratory metabolism. (**B**) In the presence of ethanol, during the growth exponential phase, the main pathway is the mitochondrial Krebs cycle, which leads to the production of energy. In the stationary phase, cells still rely on respiration, but acetyl-CoA is mainly converted to oxaloacetate through the glyoxylate pathway.

From a metabolic point of view, *S. cerevisiae* cells maintain their growth in the exponential phase by producing ATP through fermentation and/or mitochondrial respiration dependent on the type of available substrate (Figure 1).

When glucose is used as a carbon source, yeast growth is closely linked to the function of ATP-generating metabolic pathways (Figure 1A). At high glucose levels, *S. cerevisiae* prefers fermentation as the central pathway for ATP generation [5,6,22]. At low glucose levels, yeast produces ATP through mitochondrial respiration [9,22,23,24].

On the other hand, when ethanol is used as a carbon source, yeast metabolism is mainly respiratory (Figure 1B). In particular, during the exponential growth phase, the main pathway is the mitochondrial Krebs cycle, which leads to energy production [25]. When ethanol becomes limiting, yeast cells respond with growth arrest and enter the stationary phase [25,26]. During this growth phase, the yeast cells continue to produce ATP through mitochondrial respiration; at the same time, the glyoxylate pathway allows the synthesis of sugars (Figure 1B).

### 2.2. CTP1 and YHM2 Expression Levels in Wild-Type S. cerevisiae Cells Grown in Culture Media Containing Glucose or Ethanol

To understand the role of the two mitochondrial citrate transporters in the metabolic reprogramming that marks the transition from the exponential phase to stationary phase, wild-type yeast cells were grown on media containing glucose or ethanol. The transcripts of Ctp1 and Yhm2 were then analyzed at different time points of yeast growth. Since the expression levels of Ctp1 and Yhm2 did not change within the same growth phase in all strains analyzed, we chose a single intermediate time point representative of each phase (12 h for the exponential phase and 48 h for the stationary phase).

**Figure 2:**
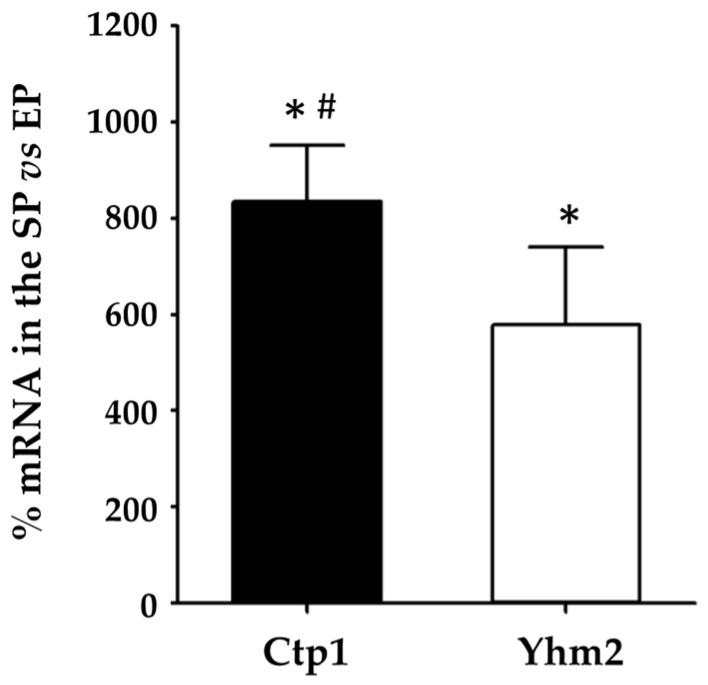
Ctp1 and Yhm2 expression in wild-type yeast cells grown in culture media containing glucose. mRNA levels in the stationary phase (SP) were compared to mRNA levels in the exponential phase (EP) which were set to 100%. * *p* < 0.005 vs. EP; # *p* < 0.005 vs. Yhm2 mRNA.

Figure 2 shows the changes in Ctp1 and Yhm2 expression levels during the transition from the exponential to stationary phase when yeast cells were grown in the presence of glucose.

We found that metabolic reprogramming (from fermentative to respiratory) was accompanied by a strong increase in the mRNA levels of both mitochondrial citrate transporters. In particular, during the stationary phase, mRNA of Ctp1 and Yhm2 increased significantly by 734% and 523%, respectively, compared with the exponential phase.

These results suggest a central involvement of the two carrier proteins in the switch of yeast metabolism to respiration, which requires an active Krebs cycle.

In parallel experiments, we evaluated the mRNA levels of Ctp1 and Yhm2 in wild-type yeast cells in the presence of ethanol as a carbon source during the exponential phase (12 h of growth) and stationary phase (48 h of growth).

**Figure 3:**
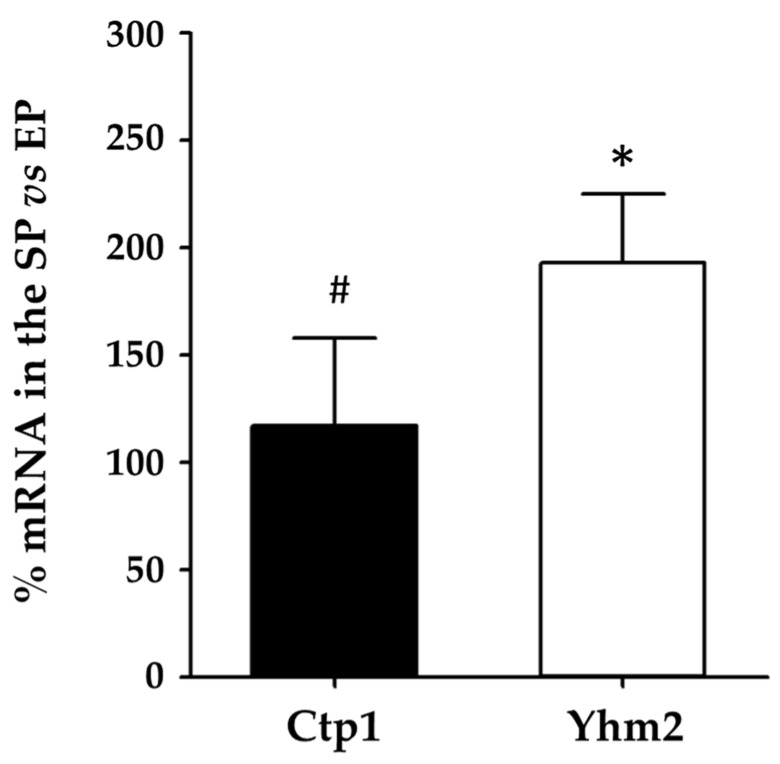
Ctp1 and Yhm2 expression in wild-type yeast cells grown in culture media containing ethanol. mRNA levels in the stationary phase (SP) were compared to mRNA levels in the exponential phase (EP) which were set to 100%. * *p* < 0.005 vs. EP; # *p* < 0.005 vs. Yhm2 mRNA.

Figure 3 shows that the mRNA levels of Yhm2 doubled compared with the exponential phase.

In this context, it is important to emphasize that the mRNA levels of Ctp1 and Yhm2 observed in the exponential phase when cells were grown in ethanol-supplemented media were approximately 8- and 3-fold higher, respectively, than the expression observed in the same phase in glucose-supplemented media.

Therefore, the results shown in Figure 3 (where mRNA levels of each carrier protein in the exponential phase were set to 100%) allow us to hypothesize that, in particular, Yhm2 plays a key role in the metabolic reprogramming of yeast associated with the transition from the exponential to the stationary growth phase when yeast cells are grown in culture media containing ethanol.

### 2.3. Effects of Deletion of CTP1 or YHM2 on S. cerevisiae Growth Behaviour

To better investigate the role of Ctp1 and Yhm2 in yeast physiology, we used two mutant yeast strains in which the gene encoding Ctp1 (∆*ctp1*) or Yhm2 (∆*yhm2*) had been deleted.

The growth rate and phenotype of *CTP1* or *YHM2* deletion strains were studied by comparing the growth behavior of mutant cells with that of wild-type cells on liquid or solid media supplemented with glucose or ethanol. This analysis was performed on yeast cells grown in both rich (YP) and synthetic minimal (SM) media. Yeast cells grow much faster in YP medium containing reagents such as yeast extract and bactopeptone. These provide many of the metabolites that the cells would synthesize when growing in an SM medium. Thus, the rich medium, which is largely sourced from crude cell extracts, does not allow us to control the amino acid composition and is therefore unsuitable for auxotrophic selection. Furthermore, when grown in nutrient-rich medium, *S. cerevisiae* cells enter a low metabolic state in which they can survive for months. In the SM medium, haploid cells maintain a high metabolic state even when nutrients are scarce, and they can survive for only about 1 to 2 weeks [27].

**Figure 4:**
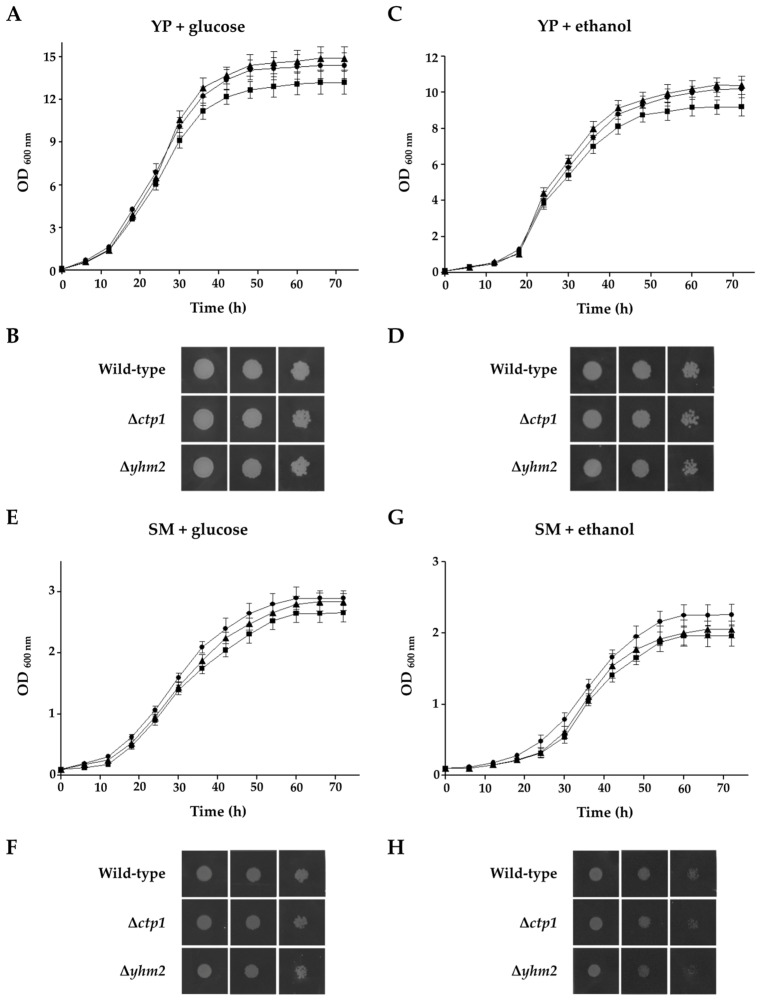
Growth behavior of wild-type and deleted strains under various conditions. Wild-type (●), ∆*ctp1* (◼), and ∆*yhm2* (▲) cells were inoculated in liquid glucose-supplemented YP (**A**), ethanol-supplemented YP (**C**), glucose-supplemented SM (**E**), or ethanol-supplemented SM medium (**G**). The optical density (OD) values at 600 nm refer to cell cultures after the indicated growth times. Three-fold serial dilutions of wild-type and deleted cells were also plated on solid YP medium supplemented with glucose (**B**) or ethanol (**D**) and solid SM medium supplemented with glucose (**F**) or ethanol (**H**). Pictures given in (**B**,**D**,**F**,**H**) were taken after 2 or 3 days to show yeast growth performance. Growth curves data are expressed as the mean ± SD. Differences were considered statistically significant for *p*-values < 0.005.

Growth curves and spot assay images are shown in Figure 4.

We found that the growth rates (0–72 h) of the three strains were quite similar when glucose or ethanol were present in the medium (Figure 4A,C,E,G). In addition, both wild-type and mutant yeast strains showed a similar growth phenotype in solid rich and minimal media supplemented with either 2% glucose or 2% ethanol (Figure 4B,D,F,H; Supplementary Figure S1). The double mutant also showed no significant growth defects, as reported in [17].

The observation that ∆*ctp1* and ∆*yhm2* yeast cells were able to grow in YP and SM media supplemented with different fermentable and nonfermentable carbon sources at similar rates to the wild-type strain (Table 1) leads us to conclude that the presence of a single citrate transporter (Ctp1 or Yhm2) can partially compensate for the absence of the other.

### 2.4. Expression Levels of Mitochondrial Citrate Transporters in Mutant Yeast Strains Grown in Culture Media Containing Glucose or Ethanol

As previously reported, *S. cerevisiae* cells exhibit a fermentative metabolic phenotype in the exponential phase in the presence of glucose as a carbon source, which switches to respiratory metabolism in the stationary phase [22,23].

To investigate whether either mitochondrial citrate transporter can compensate for the absence of the other in yeast metabolic reprogramming, we examined the mRNA of Ctp1 and Yhm2 and the protein levels of citrate transporters in the presence of glucose as a carbon source in the ∆*yhm2* and ∆*ctp1* mutant yeast cells after 12 h (exponential phase) and 48 h (stationary phase) of growth.

**Figure 5:**
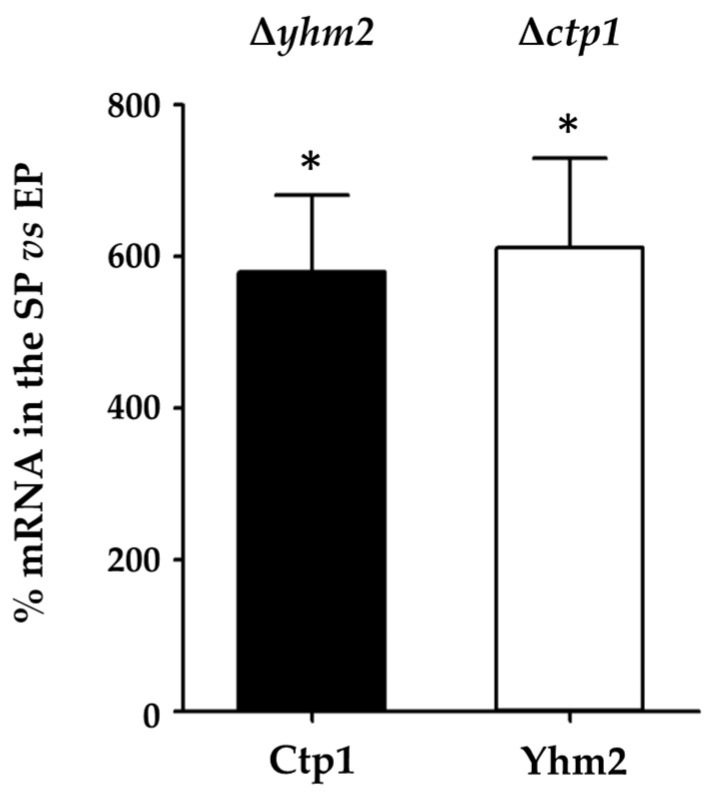
Ctp1 and Yhm2 expression in mutant yeast strains grown in culture media containing glucose. mRNA levels found in ∆*ctp1* and ∆*yhm2* yeast cells in the stationary phase (SP) were compared to mRNA levels in the exponential phase (EP) which were set to 100%. * *p* < 0.005 vs. EP.

The mRNA levels of Ctp1 and Yhm2 in the ∆*yhm2* and ∆*ctp1* strains in the stationary phase, respectively, were compared with their expression levels in the exponential phase; the results are shown in Figure 5.

We found that in the stationary phase (respiratory metabolism), the mRNA levels encoding Ctp1 (in the ∆*yhm2* strain) and Yhm2 (in the ∆*ctp1* strain) significantly increased approximately six-fold compared with the exponential phase. These results confirm the central involvement of the two carrier proteins in the switch of yeast metabolism to respiration, as already observed in the wild-type cells.

In parallel experiments, we analyzed the mRNA levels of Ctp1 and Yhm2 in mutant yeast strains in the exponential phase and in the stationary phase, in the presence of ethanol, where yeast metabolism is mainly a respiratory metabolism.

**Figure 6:**
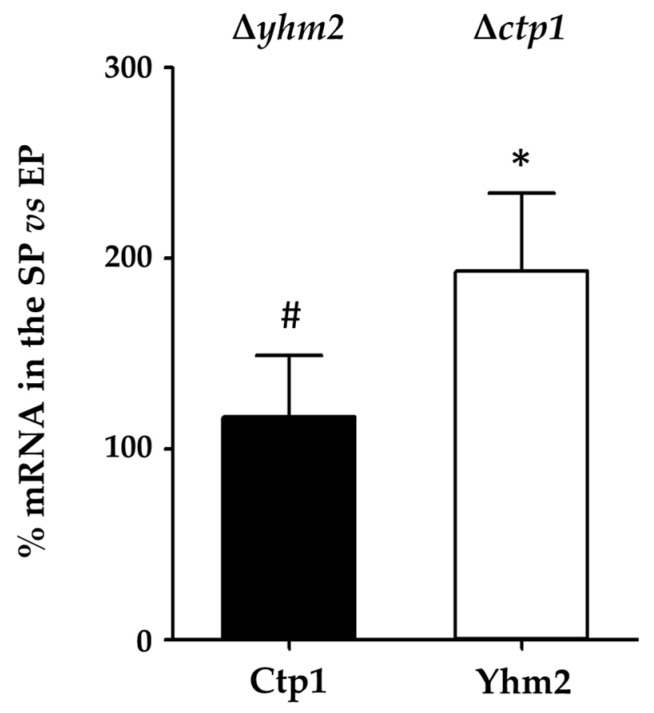
Ctp1 and Yhm2 expression in mutant yeast strains grown in culture media containing ethanol. mRNA levels in the stationary phase (SP) were compared to mRNA levels in the exponential phase (EP) which were set to 100%. * *p* < 0.005 vs. EP; # *p* < 0.005 vs. Yhm2 mRNA.

Figure 6 shows that, as already found in the wild-type strain (Figure 3), mRNA levels of Yhm2 doubled in the stationary phase compared with the exponential phase.

These results confirm our previous hypothesis that Yhm2 plays a key role in the transition from the exponential to the stationary growth phase when yeast cells were grown in the presence of ethanol.

The mRNA levels of Ctp1 and Yhm2 observed in the exponential phase when mutant cells grew in ethanol-supplemented media (respiration) were approximately 5- and 2-fold higher, respectively, than the expression observed in the same phase in the presence of glucose (fermentation).

### 2.5. Differences in Mitochondrial Citrate Transporter Expression between Mutant and Wild-Type Yeast Strains Growing in Glucose- or Ethanol-Supplemented Culture Media

The experiments performed on the mutant strains (Figure 5 and Figure 6) showed that the increase in the expression of Ctp1 or Yhm2 under the different growth conditions followed a similar trend to the wild-type cells (Figure 2 and Figure 3).

We then decided to compare the mRNA levels of Ctp1 and Yhm2 in the ∆*yhm2* and ∆*ctp1* strains, respectively, with their expression levels in the wild-type strain in the same growth phase (exponential or stationary).

**Figure 7:**
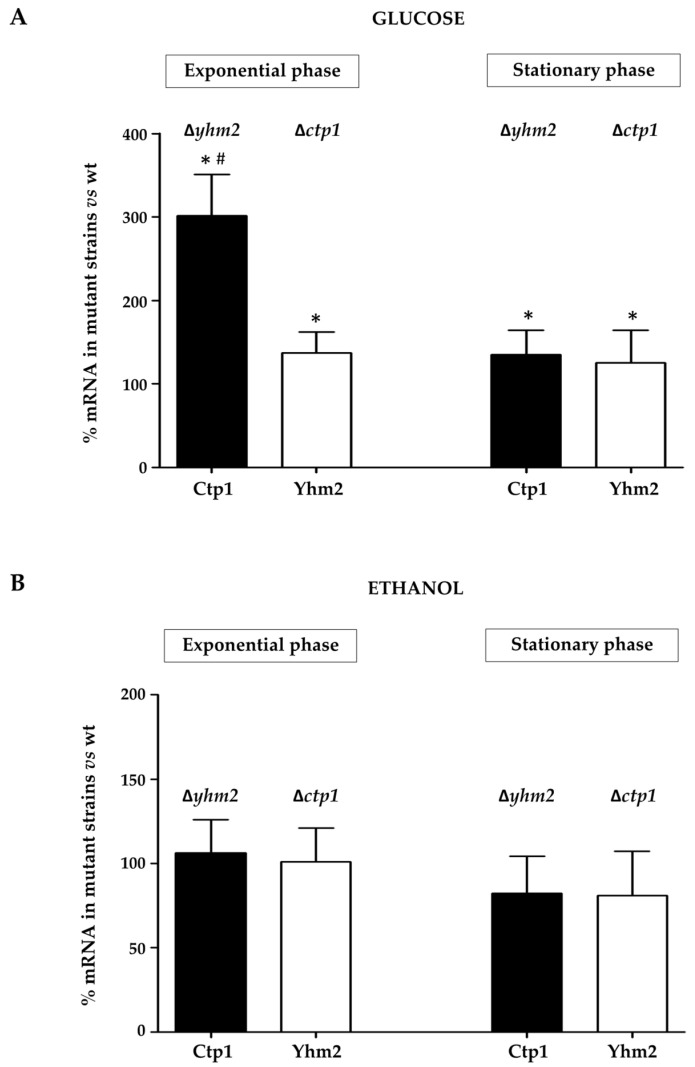
Expression of Ctp1 and Yhm2 in mutants compared with wild-type cells grown in glucose-containing (**A**) or ethanol-supplemented (**B**) culture media. mRNA levels found in ∆*ctp1* and ∆*yhm2* yeast cells were compared with the expression levels in the wild-type strain in the same growth phase (exponential or stationary), which were set to 100%. * *p* < 0.005 vs. wild-type; # *p* < 0.005 vs. Yhm2 mRNA.

When glucose was used as a carbon source (Figure 7A), we found that in the exponential phase (fermentative metabolism), mRNA levels encoding Ctp1 (in the ∆*yhm2* strain) were increased three-fold compared with the wild-type strain; Yhm2 mRNA (in the ∆*ctp1* strain) showed a significant increase of about 40% compared with the wild-type cells. The strong increase in Ctp1 mRNA levels observed in ∆*yhm2* cells after 12 h of growth in the presence of glucose suggests the hypothesis of greater involvement of Ctp1 in fermentative metabolism when Yhm2 was absent.

In the stationary phase (respiratory metabolism), the presence of one of the two mitochondrial citrate carrier proteins appears to compensate for the absence of the other. In the absence of either carrier protein, the mRNA levels of the other increased significantly by approximately 30% compared with the wild-type cells, confirming once again the central involvement of the two carrier proteins in the switch of yeast metabolism to respiration.

As described for glucose, we analyzed the mRNA levels of Ctp1 and Yhm2 in mutant yeast strains in the exponential phase and in the stationary phase, in the presence of ethanol, where yeast metabolism is mainly a respiratory metabolism.

Figure 7B shows that the absence of one of the two citrate carriers does not cause significant changes in the expression of the other.

This observation suggests that both Ctp1 and Yhm2 can transport metabolic intermediates across the mitochondrial membrane, allowing the Krebs and glyoxylate cycles to ensure cell survival on ethanol in the stationary phase, depending on the metabolic demands. Indeed, citrate can be processed in the Krebs cycle to generate reducing equivalents supporting the respiratory chain activity; at the same time, some mitochondrial citrate can replenish the glyoxylate cycle.

## 3. Discussion

In higher eukaryotes, the best-known function of mitochondrial citrate transporter is the supply of cytosol with acetyl-CoA, which is necessary for the de novo fatty acid synthesis [28,29]. Nevertheless, several additional functions have recently been proposed for the mammalian mitochondrial citrate carrier in cell metabolism and physiology [19,30].

In contrast to other species, two mitochondrial citrate transporters, Ctp1 and Yhm2, have been identified in *S. cerevisiae*. Also, in the white koji fungus *Aspergillus luchuensis* mut. *kawachii* (*A. kawachii*), which produces a substantial amount of citrate during the fermentation of shochu (a traditional Japanese distilled spirit), CtpA and YhmA have been identified as mitochondrial citrate transporters homologous to Ctp1 and Yhm2, respectively, and are mainly involved in acetyl-CoA biosynthesis [31].

However, the role of Ctp1 and Yhm2 in the yeast physiology has remained unclear. First of all, they do not appear to be associated with the transport of acetyl units in the form of citrate for fatty acid synthesis. In fact, in yeast *S. cerevisiae*, the mechanisms of cytosolic acetyl-CoA production appear to be independent of citrate transport in the cytosol. When ethanol or glucose are used as a carbon source, acetyl-CoA can be produced directly in the cytosol, so this molecule does not have to be transported outside mitochondria [32]. Furthermore, genes encoding ATP-citrate lyase (which produces acetyl-CoA from cytosolic citrate) appear absent in the non-oleaginous yeast *S. cerevisiae* [33].

In this study, we sought to elucidate the roles of Ctp1 and Yhm2 in respiration and fermentation, two metabolic processes that occur under different environmental conditions in yeast S. *cerevisiae*. To this end, we used media supplemented with glucose or ethanol as a fermentable or nonfermentable carbon source, respectively, to grow wild-type and mutant yeast cells in which *CTP1* and *YHM2* were deleted.

Experiments carried out on wild-type yeast cells grown in the presence of glucose showed that reprogramming of metabolism (from fermentative to respiratory metabolism) was associated with a strong increase in the expression of the two citrate carriers, Ctp1 and Yhm2 (Figure 2), which may contribute to the flux of citrate from mitochondria to the cytosol in a manner that supports both the Krebs cycle [34,35] and part of the glyoxylate cycle reactions [36]. Moreover, as suggested by Castegna et al., Yhm2 could also function in a shuttle mechanism citrate/α-ketoglutarate that indirectly transports NADPH from mitochondria to the cytosol [17]. Then, this protein can contribute to an increase in the cytosol NADPH reducing power, which is required for biosynthetic and antioxidant reactions.

Experiments carried out on wild-type yeast cells grown in the presence of ethanol showed that Yhm2 plays a key role in the metabolic reprogramming of yeast associated with the transition from the exponential to the stationary growth phase (Figure 3). The large increase in the expression of this citrate transporter may be due to the role of this carrier in the production of NADPH, which is useful to counteract the production of reactive oxygen species (ROS) whose levels increase with cell growth and aging [37], particularly in the presence of nonfermentable carbon sources that require higher mitochondrial respiratory chain activity.

To better investigate the role of Ctp1 and Yhm2 in yeast physiology, we used the two mutant yeast strains ∆*ctp1* and ∆*yhm2*, in which the gene encoding Ctp1 or Yhm2, respectively, had been deleted. These strains were found to be capable of growing on ethanol and glucose, as reported in this (Figure 4) and previous studies [16,17,38,39], indicating that the presence of a single citrate transporter (Ctp1 or Yhm2) can partially compensate for the absence of the other.

We found that in the presence of glucose as a carbon source, in the stationary phase (respiratory metabolism), the expression of Ctp1 (in the ∆*yhm2* strain) and of Yhm2 (in the ∆*ctp1* strain) significantly increased compared with the exponential phase (Figure 5). This result confirms the central involvement of the two citrate transporters in the metabolic switch to respiration, which requires an active Krebs cycle. Moreover, the comparison with wild-type cells, which shows a large increase in Ctp1 mRNA levels in ∆*yhm2* cells after 12 h of growth (Figure 7A), suggests the hypothesis of greater involvement of Ctp1 in fermentative metabolism when Yhm2 is absent.

To justify the sharp increase in Ctp1 expression in the absence of Yhm2, it could be hypothesized that Yhm2, unlike Ctp1, is more flexible in transporting different substrates in counter transport with citrate to better meet the biosynthetic needs of cells. Therefore, to compensate for its absence, Ctp1 must have a higher transport activity. In accordance with this hypothesis, analysis of the literature shows that Ctp1 has a stricter substrate specificity for tricarboxylic acids, transporting only citrate and isocitrate; malate can be also transported, albeit to a much lesser extent [16,40,41,42]. Yhm2 seems to be able to transport citrate using a wider variety of counter substrates, including citrate, α-ketoglutarate, oxaloacetate, succinate, and fumarate [17].

When yeast mutant strains were grown on ethanol as a carbon source, we found that the absence of either citrate carrier does not cause significant changes in the expression of the other compared with expression levels in the wild-type strain in the same growth stage (Figure 7B). These results suggest that both Ctp1 and Yhm2 can transport metabolic intermediates from the mitochondria to the cytosol and vice versa, allowing the Krebs and glyoxylate cycles to ensure cell survival in the stationary phase.

In conclusion, apart from the specificity of Yhm2 in the indirect production of NADPH [17], the results of this study suggest that the two citrate transporters in yeast provide a link between the Krebs cycle and the glyoxylate cycle reactions. Because these metabolic pathways play a key role in the metabolic adaptation strategies of *S. cerevisiae*, the transport of citrate and other metabolic intermediates has a central role in linking metabolic reactions between the cytosol and mitochondria.

Understanding yeast metabolism could also have an impact from a clinical point of view. In this context, it has been reported that *S. cerevisiae* has been recently studied for its probiotic function as a treatment strategy for relieving intestinal imbalance. In particular, a recent study demonstrated that *S. cerevisiae* has a probiotic effect by improving intestinal immune homeostasis and function and reducing systemic and colonic inflammation, suggesting the possibility of developing it into a probiotic yeast [43]. Importantly, a potential role of *S. cerevisiae* and mucosal immunological response to *S. cerevisiae* has been described also in autoimmune liver [44] and intestinal disease, such as celiac disease, where a significant percentage of patients develop anti-*S. cerevisiae* antibodies as a consequence of an increased intestinal permeability [45]. This evidence suggests that the study of yeast metabolism could also be useful to investigate a potential probiotic therapeutic intervention as adjunctive therapy in autoimmune liver and intestinal diseases, as recently suggested [46].

We are aware that our analysis represents only one aspect of the complex network of metabolic pathways in which Ctp1 and Yhm2 may be involved. Indeed, a true functional or physiological differentiation is not possible based on the preliminary data presented in this manuscript. Therefore, further research is needed to gain new insights into the physiological role of mitochondrial citrate carriers in cell metabolism of *S. cerevisiae*, given the exceptional importance of this model organism. In this context, it is crucial to analyze the double deletion mutant lacking Ctp1 and Yhm2 to identify redundancies or differences between the two citrate transporters. Direct studies of the transport activity into isolated mitochondria could also be useful to further clarify aspects related to the physiological roles of these two citrate carriers.

## 4. Materials and Methods

### 4.1. Yeast Strains and Growth Conditions

*S. cerevisiae* strains BY4742 (Matα; his3Δ1; leu2Δ0; lys2Δ0; ura3Δ0), ∆*ctp1* (Matα; his3Δ1; leu2Δ0; lys2Δ0; ura3Δ0; YBR291c::kanMX4), and ∆*yhm2* (Matα; his3Δ1; leu2Δ0; lys2Δ0; ura3Δ0; YMR241w::kanMX4) were provided by EUROSCARF (Frankfurt, Germany). The wild-type and deleted strains were grown at 30 °C in rich medium (YP), containing 2% (*w*/*v*) bactopeptone and 1% (*w*/*v*) yeast extract, or in synthetic complete medium (SM), containing 0.67% (*w*/*v*) yeast nitrogen base (Difco) and 0.1% (*w*/*v*) drop-out mix. The optimal pH range for yeast growth can vary from pH 4.0 to 6.0. So, we used pH 4.8 for YP and pH 4.5 for SM media [47]. For solid media, 2% *w*/*v* agar was used [48].

Growth curves of yeast strains were monitored in rich medium (YP) or in synthetic minimal (SM) medium, supplemented with a fermentable (2% *w*/*v* glucose) or a nonfermentable (2% *v*/*v* ethanol) carbon source, using a benchtop orbital shaking incubator (Thermo Scientific™ MaxQ™ 4000, Waltham, MA, USA) with constant agitation at 120 rpm in 50 mL flasks for 72 h. Briefly, yeast strains were precultured overnight in YP supplemented with 2% glucose to early stationary phase and transferred, after washing, to the media to a final optical density (OD) of 0.1 at 600 nm. The OD at 600 nm was measured using a spectrophotometer (Jenway™ 7200, Fisher Scientific, Hampton, NH, USA) at regular time intervals. All strains were cultured in triplicate. Simultaneously, cell concentration was determined by cell counting in a Bürker chamber, and cells were washed, diluted, and spotted on solid glucose-supplemented YP, glucose-supplemented SM, ethanol-supplemented YP, or ethanol-supplemented SM medium. Then, plates were incubated for 48 or 72 h at 30 °C. Three-fold serial dilutions of wild-type, ∆*ctp1,* and ∆*yhm2* strains were analyzed [49].

Kinetic parameters (growth rates and doubling times) were calculated by using GraphPad software (GraphPad Prism version 8.0.2 for Windows, GraphPad Software, Boston, MA, USA) [24].

### 4.2. RNA Extraction and Real-Time Quantitative PCR Analyses

For preparation of total RNA, the wild-type and deleted strains were precultured overnight in YP supplemented with 2% glucose to early stationary phase and reinoculated, after washing, in fresh medium (YP), supplemented with a fermentable (2% *w*/*v* glucose) or a nonfermentable (2% *v*/*v* ethanol) carbon source, to a final OD of 0.1 at 600 nm. Total yeast RNA was isolated from exponential-phase cultures or stationery-phase cultures. Briefly, 5 × 10^7^ yeast cells per sample were harvested by centrifugation at 4000× *g* for 10 min at room temperature and the pellets were washed in 1 M sorbitol, 0.1 M EDTA. Cell lysis was then performed by incubating cells with 25 U/mL zymolyase 20 T (from Arthrobacter luteus, MP Biomedicals, Santa Clara, CA, USA) at 30 °C for 30 min. Then, total RNA was extracted from yeast spheroplasts using the Blood/Tissues Total RNA Extraction Mini Kit (Fisher Molecular Biology, Rome, Italy) following the manufacturer’s protocol. The RNA concentration and purity were estimated using Nanodrop™ (Thermo Scientific™, Waltham, MA, USA) spectrophotometer, by evaluating the A_260_/_280_ ratios (of approximately 2.0).

One microgram of total RNA was reverse transcribed into first-strand cDNA in 10 µL reaction using PrimeScript^TM^ RT Master Mix (TaKaRa, Kusatsu, Japan), a pre-mixed reagent containing all the components needed for quantitative RT-PCR reverse transcription (PrimeScript RTase, RNase Inhibitor, Random 6 mers, Oligo dT Primer, dNTP Mixture, and reaction buffer, TaKaRa, Kusatsu, Japan). The cDNA synthesis was performed in a Mastercycler^®^ personal (Eppendorf, Germany) using the following program: 37 °C for 15 min, 85 °C for 5 s. The cDNA concentration and purity were estimated by evaluating the A_260/280_ and the A_260/230_ ratios (of approximately 1.8 and 1.8–2.2, respectively) using Nanodrop™ spectrophotometer.

To assess changes in Ctp1 and Yhm2 mRNA expression levels, real-time PCR was performed on StepOne™ Real-Time PCR (Applied Biosystems, Invitrogen, Waltham, MA, USA) instrument using TaqMan^®^ Gene Expression Master Mix (Applied Biosystems) and TaqMan^®^ Gene Expression Assays (Applied Biosystems). TaqMan Gene Expression Master Mix contains AmpliTaq Gold DNA Polymerase, UP (Ultra Pure, Darien, CT, USA), a blend of dNTPs with dTTP/dUTP and uracil-DNA glycosylase (UDG) to minimize carry-over PCR contamination, and a passive internal reference based on proprietary ROX dye. Each TaqMan^®^ Gene Expression Assay consists of a fluorogenic FAM™ dye-labeled MGB probe and two gene-specific amplification primers (forward and reverse) provided in a preformulated 20× mix; 1× final concentrations are 250 nM for the probe and 900 nM for each primer (*CTP1*, Assay ID:Sc04103876_s1; *YHM2*, Assay ID:Sc04156056_s1; *ACT1*, Assay ID:Sc04120488_s1). The TaqMan^®^ Gene Expression Master Mix and Assays were combined according to the manufacturer’s protocol, and the appropriate volume of the PCR Reaction Mix was transferred to each well of a 48-well standard plate. cDNA template (140 ng in nuclease-free water), or nuclease-free water for NTC (no template control), was added to each well. Thermocycling conditions consisted of UNG incubation of 2 min at 50 °C and an initial denaturation of 10 min at 95 °C, followed by 40 cycles at 95 °C for 15 s and 60 °C for 60 s. Relative quantification of gene expression was performed according to the ΔΔ−CT method and *ACT1* gene was used as an internal control for normalization [50]. The mRNA levels in cells in the stationary phase are expressed as % of the mRNA levels in exponentially growing cells.

## 5. Conclusions

**Figure 8:**
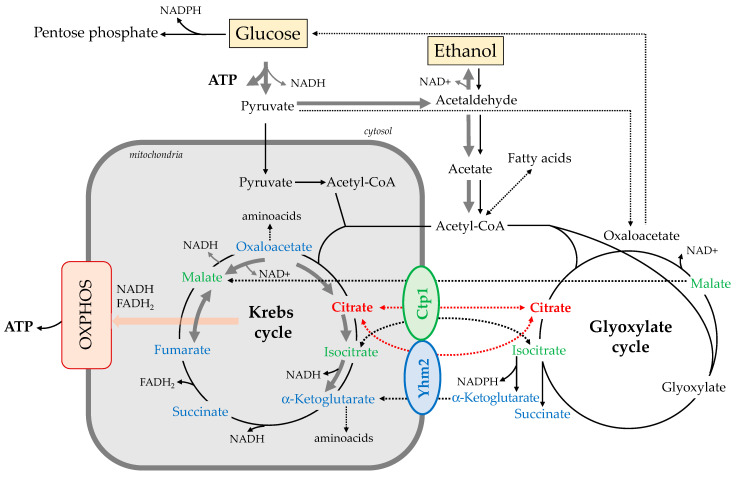
Role of Ctp1 and Yhm2 in yeast metabolism in the presence of fermentable (glucose) and nonfermentable carbon sources (ethanol). The transport of citrate is shown in red. Substrates that can be transported by Ctp1 are colored green, while those that can be transported by Yhm2 are colored blue. Grey and black arrows describe fermentative and respiratory metabolism, respectively.

The aim of this study was to clarify some aspects of the physiological role of the two citrate transporters in *S. cerevisiae* that have been studied less. Our results suggest that Ctp1 and Yhm2 contribute to fermentative and respiratory metabolism in different ways and also provide an important link between the Krebs cycle and the glyoxylate cycle (Figure 8).

Although many aspects remain to be elucidated, this study provides the first evidence that Ctp1 and Yhm2 support metabolic flexibility in *S. cerevisiae* in the presence of fermentable and nonfermentable carbon sources.

## Figures and Tables

**Table 1:** Growth rate and doubling time of wild-type and mutant cells grown in YP and SM media in presence of glucose or ethanol as a carbon source. Growth rate data are the mean ± SD of three independent experiments, each performed in triplicate.

	Growth Rate (h^−1^)				Doubling Time (h)			
	YP + Glucose	YP + Ethanol	SM + Glucose	SM + Ethanol	YP + Glucose	YP + Ethanol	SM + Glucose	SM + Ethanol
Wild-type	0.408 ± 0.037	0.300 ± 0.026	0.089 ± 0.006	0.064 ± 0.004	1.69	2.33	7.77	10.84
Δ*ctp1*	0.421 ± 0.036	0.306 ± 0.025	0.088 ± 0.007	0.060 ± 0.004	1.64	2.29	7.85	11.39
Δ*yhm2*	0.437 ± 0.039	0.316 ± 0.027	0.087 ± 0.007	0.058 ± 0.004	1.58	2.22	7.90	11.76

## Data Availability

The datasets generated and analyzed during this current study are available from the corresponding author upon reasonable request.

## Supplementary Materials

The following supporting information can be downloaded at: https://www.mdpi.com/article/10.3390/ijms25031870/s1.
