# Supplementary material for: Ctp1 and Yhm2: Two Mitochondrial Citrate Transporters to Support Metabolic Flexibility of Saccharomyces cerevisiae
Source: Int J Mol Sci. 2024 Feb 3;25(3):1870. doi: 10.3390/ijms25031870 (PMC10855732; doi:10.3390/ijms25031870)
Supplement: Supplementary file 1 [file ijms-25-01870-s001.zip › ijms-2802655-supplementary.pdf]

International Journal of
Molecular Sciences

MDPI

Figure S1

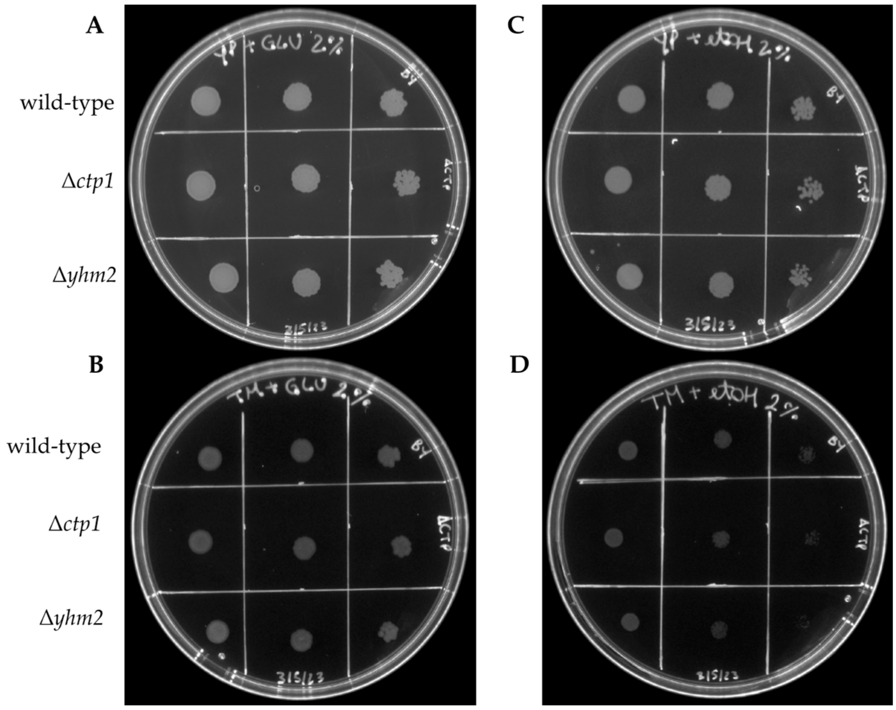

Figure S1. Growth behaviour of wild-type, Δ*ctp1* and Δ*yhm2* under various conditions. Three-fold serial dilutions of wild-type and deleted cells were plated on solid YP medium supplemented with glucose (**A**) or ethanol (**C**) and solid SM medium supplemented with glucose (**B**) or ethanol (**D**).
